# “Always Look at the Clock”: Psychosocial Working Conditions in Outpatient Care—A Qualitative Study

**DOI:** 10.3390/healthcare11233043

**Published:** 2023-11-26

**Authors:** Monika Bernburg, Volker Harth, David A. Groneberg, Stefanie Mache

**Affiliations:** 1 Institute of Occupational, Social and Environmental Medicine, Goethe University Frankfurt, 60590 Frankfurt, Germany; monika.bernburg@gmail.com (M.B.); groneberg@med.uni-frankfurt.de (D.A.G.); 2 Institute for Occupational Medicine and Maritime Medicine (ZfAM), University Medical Center Hamburg-Eppendorf (UKE), 20459 Hamburg, Germany; harth@uke.de

**Keywords:** outpatient nursing personnel, stress factors, resources, working conditions, mental health, health promotion

## Abstract

Background: In view of demographic change, there is a growing need for qualified nursing professionals. More and more people in need of care prefer to be cared for at home. A shortage of skilled workers and constantly changing and increasing professional requirements are some of the consequences. The aim of this study is to generate further insights into work-related psychosocial stress factors and the resources of outpatient caregivers in their subjective perceptions to derive approaches for possible health-promoting interventions for practice and research that promote healthier and more sustainable work. In addition, coping strategies and needs should be inquired about in order to determine support offers. Methods: Using a semi-structured interview guide, thirty outpatient nursing professionals in Germany were interviewed in 2022. The subject of this interview was the psychosocial working conditions and resources in outpatient care. The collected data were evaluated by means of the Kuckartz qualitative content analysis. Results: This qualitative study identified work-related job demands and resources in outpatient care. The way nurses are treated in the companies they work for and the appreciation of patients were explored as important resources. Physical demands, the time factor, and dealing with personal fates are primarily mentioned as stress factors. The learning effect plays an important role in dealing with these. Conclusion: This study contributes to a better understanding of current job strain and resources as well as job satisfaction in outpatient care. In any case, it became clear that the nursing staff love their profession, which they see more as a vocation. Future quantitative studies are necessary to build on these findings. First ideas of implications for health promotion measures in research and practice have already been derived.

## 1. Introduction

### 1.1. Outpatient Care: An Increasing Demand in Germany

The number of people in need of care in Germany is growing continuously [1,2,3]. According to forecasts, 4.4 million people will be in need of care in Germany in 2050 [4]. This is accompanied by a growing demand for qualified nursing staff in inpatient and outpatient care for the elderly and sick [1,2,3,5]. The majority of people in old age want to be able to stay in their own home for as long as possible, increasing the focus on outpatient care. Outpatient care gives the person to be cared for the opportunity to stay in their own home. The place of residence and living of people in need of care thus becomes the focus of consideration [6,7,8,9,10]. In addition, demographic changes and the associated shift in age structures are more present in society than ever before [2,11,12]. This is accompanied by the following major challenges [2]:

- Greater demand for skilled workers in the care professions due to the growing proportion of people over 65;
- Simultaneous decline in the number of people of working age who could take over care.

One of the consequences of this is a growing shortage of skilled workers in the nursing profession, particularly in the care of the elderly and sick [3,5,11,12]. The current working and employment conditions of skilled workers in the care professions represent an additional challenge. The focus here is on physical and psychological stress factors. On the one hand, these strains are classified as too high compared to the relatively low pay of nursing staff. On the other hand, the demands for flexibility, especially in outpatient care, and the low attractiveness of the profession in the eyes of the next generation are linked to them [5,13,14,15]. In 2018, the number of days of incapacity to work in the nursing professions was 22.9 in comparison to 14.9 for the working population as a whole [16]. This is mainly due to psychiatric and musculoskeletal disorders, which are likely to increase in the future [2]. As a result, new strategies are needed to enable workers to remain in care professions as long and as healthily as possible [2]. It is therefore becoming increasingly important to offer health promotion and prevention measures in order to maintain the health of the nursing staff [17,18] and thus to be able to adequately cover the need for care [19,20]. Against this background, more attention should be paid to the design of the work situation, the work itself, and organisational tasks [6].

Up to now, research studies, particularly in the international area, have focused primarily on the general and inpatient nursing sectors [11,20,21,22]. In Germany, outpatient care has so far received only marginal attention in terms of subjectively perceived working conditions in science and practice [22].

### 1.2. Theoretical Framework

The Bakker and Demerouti (2007) Job Demands-Resources (JD-R) model [23] acts as a theoretical framework and can help to characterise and analyse the current work situation of outpatient nurses in order to develop target-group-specific measures. While other models often focus on only work demands or work resources, the JD-R model combines both approaches, and can thus be seen as an integrative model that embodies a dynamic character [23,24,25]. The aim of the tool is to provide a comprehensive picture of the work demands and resources of outpatient care workers and to identify factors that promote or prevent stress [26]. In terms of practical relevance, it can help in the development and implementation of work design measures. The focus here is on increasing work commitment and reducing the consequences of stress [25].

In the JD-R model, work demands include physical, mental, social, and organisational work elements that require work effort and thus have an impact on mental as well as physical health. Examples of work demands are time, content, or emotional challenges (e.g., high work pressure or emotional strain) [23,24,25,27]. Although the work demands can initially be value-neutral and not negative, they may be transformed into unfavourable and therefore stressful demands. This happens when employees are not able to balance the demands of work with coping strategies. In the course of this, work demands can be predictors of burnout and, indirectly, of the length of absence, thereby triggering health impairments [23,24,25,27,28]. According to the Joint German Occupational Health and Safety Strategy (GDA), psychological stresses can arise from the areas of work content, work organisation, social relationships, the working environment (spatial and social), and new forms of work or from their interactions [15,29]. The deciding factor for the positive or negative effect of an initially neutral burden is the individual situation of each person [30], meaning it depends on the respective individual resources and conditions. These effects are defined as “stress” or “stress sequence” [29]. Mental stress is the direct effect of mental pressure on the individual, depending on his or her respective persistent and current prerequisites, including individual coping strategies [31]. This stress is influenced by various factors (e.g., behaviour, state of health, age, attitude, and experience), which are different for each person. This is the reason for the individual and heterogenic reactions [29,32,33]. According to the GDA, the stresses are broken down into short-term positive (e.g. activation) and negative (e.g., fatigue) as well as long-term positive (e.g., exercise and further development) and negative consequences (e.g., absenteeism and burnout) [29,34]. If the stress continues over a longer period of time, long-term negative consequences for the health of the employees and the company may follow [15]. Psychological stress can be motivating and, if successfully mastered, can strengthen self-confidence or be overburdening, triggering anxiety and physical tension [29]. The five characteristic areas mentioned above also offer the starting points for work design, which not only avoids or reduces the negative consequences of stress but can even lead to positive stress consequences and thus support, promote, and secure the health of employees in the long term [29].

Coping with these different demands is at the centre of every life. These demands include interpersonal, social, and physical–environmental aspects. In addition, they are requirements resulting from the biological, psychological, and social needs of human beings and from the objectives developed by human beings themselves [35]. The means, characteristics, and conditions available to humans, also referred to here as resources, are of great importance in order to be able to cope with these demands [35,36]. 

The JD-R model also uses this in the context of the occupation. Resources are essential to compensate for work demands, achieve work goals, and trigger work motivation [23,24,25,27,28]. Work resources represent the physical, psychological, social, and organisational working conditions that stimulate personal development, reduce psychological and physical impairments, and have a positive influence on the achievement of work-related goals [23,24,25,27]. Resources are thus personal, social, and material circumstances and objects, means, and characteristics that the individual can use to cope with the external and internal demands of life and goals [35]. It depends on the individual prerequisites and circumstances what exactly can function as a resource [35]. Work resources can be at the level of the whole organisation (e.g., salary), work tasks (e.g., task diversity) and their consequences, work organisation (e.g., participation in decision making), social relations (e.g., team climate), and work demands [23,24,25,27].

Through the actively acting individual and the associated transformation of the workplace and activities, the JD-R model can be used to maintain the motivation and health of employees [24,25,28]. Personal resources include positive self-assessment and the ability to successfully control and influence the environment. Personal resources are expected to mitigate the undesirable effects of work demands on stress and to increase the desirable effects of (challenging) job demands on motivation [24,28].

In summary, it can be said that work demands and resources interact with each other and thus have a multiplier effect on the well-being of employees [23,24,28]. In addition, the working conditions, health, and motivation of employees also influence each other. For example, health and motivation also influence the working environment of employees.

### 1.3. Study Aim

The aim of our study was to examine the current work-related mental stress factors and resources in outpatient care in Germany. It is intended to provide insight into the working conditions and the health of outpatient care workers. 

Regarding the care of people in need, outpatient care is increasingly becoming the focus of attention. The aim of research and practice should be to secure skilled workers in this field; to strengthen their health, employability, and job satisfaction; and to increase the attractiveness of the profession [5]. This results is an urgent need for prevention [15]. The challenges described above make it necessary for the healthcare system to adapt to the structural changes mentioned. Politicians and stakeholders must be sensitised to the challenges of demographic development. A variety of activities, including social and individual prevention and rehabilitation or the allocation of resources, are needed [37]. Health promotion and prevention measures could contribute to strengthening professionals, and especially nursing staff, in the outpatient sector [6].

A healthy workplace design is needed in which stress is avoided and resources are not only the responsibility of the employees but also that of the employers, and thus the synthesis of both actors is achieved [30]. To be able to develop these measures specifically for the target group, it is important first to identify the work-related mental stress and resources of the nursing staff working on an amicable basis, as well as the weighting of these factors. In addition, their interrelationships must be analysed. These well-founded results could form the basis for a target-group-specific development of health promotion measures. Although there are already some studies available on research into the working conditions of outpatient nursing staff, there are still numerous gaps in the understanding of current workloads and resources in outpatient care for the elderly and sick and their interaction, particularly in Germany and against the background of changing social structures and the working world. Therefore, the present research project takes a more concrete look at the following research question: 

“*Which work-related mental stress factors and resources are perceived by outpatient nursing staff*?”.

## 2. Methods

### 2.1. Study Design

In context with the topic and the research question, the qualitative method of guideline-based interviews was selected for this work. A qualitative research design is needed for sensitive recording and differentiation between different topics, such as the specific influence of normative assumptions, subjective theories, or the reconstruction of forms of everyday knowledge [22,38,39]. Qualitative interviews focus on the individuality of a person [39] and offer the interviewees a large space in which to respond [38].

The interviewer’s previous experience with guideline-based interviews contributed to a confident appearance and the creation of a pleasant atmosphere [40]. Thirty interviews were conducted in a personal meeting.

### 2.2. Study Sample and Recruitment of Participants

The target group of the research project is outpatient nursing staff in Northern Germany. In order to comply with the idea of a target group with few selective characteristics, the interview partners were recruited from different care services in Northern Germany. Recruitment of the study participants took place via the snowball procedure in the research community. In addition, recruitment was carried out using the gatekeeper principle. Here, key persons in the company (e.g., from the human resources department) take the initiative to recruit employees from the company for an interview [40]. Thus, the care services from different districts were subsequently contacted by e-mail and/or telephone. The attempt to form a sample that covers the heterogeneity of the field corresponds to the quality criterion of external validity in qualitative research [40,41]. 

The inclusion criteria were the following: The outpatient nursing staff must have sufficient knowledge of German to understand and be able to answer the questions of the interview. In addition, they should have been working in small and medium-sized enterprises (SME) for at least six months. The SME criterion was defined because hardly any health promotion measures exist in these companies, and the need is therefore greatest there. In most cases, the necessary financial, structural, and human resources are not available to be able to expand these measures [42,43].

Study participation was voluntary for the outpatient caregivers. Each participant was asked to sign a declaration of informed consent regarding performance and recording prior to the interviews. All interviewees were in a position to understand and to consent to the study requirements, and they were provided written informed consent. 

### 2.3. Interview Guideline and Data Collection

A semi-structured interview guide was designed within the general framework of the empirical and theoretical background. The guideline was tested in several pre-tests. Questions on the following categories were asked:

1. Introduction;
2. General information;
3. Working activity;
4. Stress factors at work;
5. Health impairment;
6. Support needs;
7. Work resources;
8. Wishes and needs;
9. Closing.

The interview guide was tested beforehand to receive feedback from colleagues and nurses to check the duration of the interview and the comprehensibility of the questions. The topics job demands (e.g., perceived work intensity) and job resources (e.g., perceived positive, supportive characteristics at work) were included in the interview guide.

An appointment could be made with thirty interview partners from ten different nursing services. The interview partners fulfilled all the inclusion criteria described above. The interviews were recorded with an audio device and then transcribed [38,44]. The interviews were conducted in German and averaged 40 min in length. The interviews were then transcribed using the audio recordings, which formed the basis for the data evaluation.

### 2.4. Data Analysis

As the evaluation methodology of the data material within the framework of this research project, the qualitative content analysis according to Kuckartz was chosen [45]. It is a frequently used and proven method that is convincing because of its simplicity [45,46,47]. Kuckartz [45] focuses in particular on the category-based evaluation phase of guideline-oriented interviews and the presentation of results, as was also planned for the research project mentioned here [46]. Based on the research question, the first step was to work on the text using transcriptions of the interviews recorded on audio. The transcript should give the researchers and readers the best possible impression of the interview, but too many details lead to poor readability. The transcription was carried out using the f4transcript programme.

The category formation was based on the guidelines and, thus, on the literature and the JD-R model. Since the research project requires the examination of a theoretical framework, to which the elaborated guidelines also refer, categories could be formed even before the evaluation of the collected data. Therefore, the so-called a priori categorisation (deductive categorisation) was applied in this project. However, after the first rough categorisation, differentiation in the form of inductive categorisation was also applied to the project [45]. The reason for this was that during the evaluation of the data material and based on initial findings, main categories and sub-categories still emerged. For this reason, deductive–inductive categorisation and coding—a hybrid form—were applied. MAXQDA 2020 (VERBI Software, 2019, VERBI GmbH, Berlin, Germany) was used for the data analysis. To guarantee a high quality of data evaluation, the category system was successively adapted during the evaluation process.

## 3. Results

### 3.1. Sample Characteristics

A total of 28 women and 2 men were interviewed. The interview partners are between 23 and 61 years old. The average age is 47 years. All of them have German nationality. Five nurses have a baccalaureate degree. Twenty-one of the thirty interviewees are qualified nurses and healthcare professionals. Twenty-eight interviewees travel to work via a car, and two participants travel by bicycle. The interviewees mainly work the early and late shifts. Six caregivers have a 30 h work week. Some nurses have already gained experience in other outpatient nursing services or in an inpatient facility—training was excluded. 

### 3.2. Job Demands

**Figure 1:**
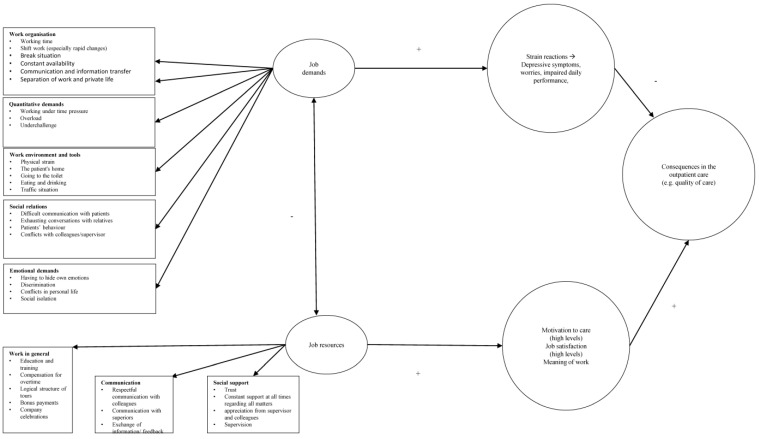
Job demands, resources and work-related health outcomes in outpatient care.

In adherence to the JD-R Model, the results of the interviews are visualised in Figure 1. Within the workloads, which were divided into sub-categories, the time factor, quantitative demands, and dealing with the patients’ personal fates were particularly mentioned on the part of the nurses. Emphasis was placed on the fact that the stresses and strains were particularly present when starting work. In the course of time, they would no longer think about them, or they would not even be aware of them.

#### 3.2.1. Work Organisation

In the area of work organisation, the time factor was the most frequently cited stress factor by almost all outpatient nursing staff. Especially at the start of their careers, time was a burden, and the nursing staff first had to learn how to deal with it, or in some cases, it was still present. This was always in the back of their minds.

*“[…] always look at the clock a little bit and then you’re always doing the calculations, so okay, you don’t need that long, so that’s how it is, it’s often an example of calculation, as far as time is concerned. Time is of course our biggest enemy, actually”*

(I1, A49–50)

#### 3.2.2. Work Content

In the area of work content, it was described that mental power in work as an outpatient nurse was required. Many outpatient carers argued that you never know what is going to happen to old people. Something unexpected can always happen, which means that nurses need to be able to make decisions in critical situations on their own, to react quickly, and to constantly reassess patients. You can never be prepared for everything. But the longer the job is performed, the better these situations can be managed. However, some situations can still come as a surprise, despite many years of professional experience.

*“[…] yes, you always don’t know what’s hiding behind the next door. That’s always the case, especially when you know, uh, yesterday morning the lady was not feeling well, what’s today, is she dead in bed or is a, you don’t know, that’s also the case, where you always have a bit of, hmpf (...) which is always not very pleasant and hmpf otherwise (...)”*

(I4, A63–64)

In addition, it is a great emotional challenge for all nursing staff to deal with the personal fates of patients and to distance themselves from them. This is particularly difficult against the background of increasing sympathy for the patients. Carers described that dealing with suffering and dying people has a strong impact on the psyche. The restlessness of not knowing that the patient is being well looked after, for example, is also a burden. At the same time, carers argue that coping with suffering and dementia is something that has to be learned and that it is normal that this, especially in the beginning, can be challenging.

#### 3.2.3. Work Environment

In the context of the stress factors associated with the working environment, the physical strain was primarily mentioned by outpatient nursing staff. The consensus is that their work is highly physical work and that it places strain on the back. It is exhausting to be on the move all day. This is particularly noticeable with increasing age. There are few carers who feel that these strains are less severe. The outpatient nursing staff are concerned that work is no longer affordable in old age because of the physical strain—not the emotional challenges.

*“Yes, I don’t want to stop at all (laughs). I don’t want to retire at all (laughs). I have always been asking what I have to do so that I don’t have to retire, how does Merkel do it when she’s over 65 (laughs). [...] No, um (...) yeah, but um, I think it is rather the physical limitations that will prevent me from doing so. So I have already realised what it is that prevents me from doing so, yes”*

(I12, A107–108)

### 3.3. Job Resources

In the area of labour resources, which were structured according to organisational, social, and personal aspects, the following aspects can be observed in particular: the great love of the profession, among other things due to the high degree of autonomy, flexibility, and independence; the sufficient appreciation by the companies in various forms; the good relationship between colleagues; and, as the greatest motivation, the gratitude of the patients. The results are visualised in Figure 1.

#### 3.3.1. Personal Resources

Regarding the resources on a personal level of the interviewed outpatient nursing staff, it can generally be observed that they love their profession. They stated that they carry out their work with one hundred percent effort.

*“[…] what I always find important is that you put your heart into it and you really want to do it, because you simply work very closely with people and yes […]”*

(I4, A159–160)

The nursing staff are simply enthusiastic about outpatient care. The interview partners appreciate the independent and autonomous work and the freedom and flexibility that come with it. They all find their work very meaningful, and they find it positive to be able to help people.

#### 3.3.2. Organisational Resources

As far as organisational resources are concerned, it should be noted that many outpatient nursing staff are very satisfied with several aspects of work organisation. It is noticeable that when describing organisational resources, they compared their current situations to other care services where they had to work under different conditions that they described as worse. As a result, the carers have already had other experiences that they did not like and with which they were not happy. Compared to their current employers, there were big differences, such as previous permanent replacements, working without aid or any kind of support, constant changes in the patient base, and very strict time limits, which meant that the fun in the work was lost. Perhaps they only notice the contrast so strongly because they know and are used to the differences, according to the interview partners. In the following, an outpatient nursing staff member states that they receive a lot of appreciation from the current company on several levels. They are treated very well and taken seriously, which makes them feel good.

*“Yes, 10 (laughs). No, I am very satisfied here at the company now. Everything is organised, it’s very familiar here, it’s (take a deep breath) when information comes, always with a little heart, and it’s really great, so I really have to say it’s nice, it’s smaller than what I know, but it’s just very familiar”*

(I4, 129–130)

#### 3.3.3. Social Resources

##### Family and Friends

Some carers noted that they do not experience conflict between their work and private lives and that they receive full support from their family or partners. The support of family and friends is still very important to create a good balance between work and private life. 

##### Colleagues and Supervisors

Regarding colleagues, all the interviewees made it clear that they have a very good (trusting) relationship with them. Most of the interviewees get along very well with their colleagues and exchange information regularly, and they help each other in the form of tips, advice, and experience, which almost all of them consider very important and valuable.

*“ALWAYS, the [exchange] always prevails. It’s guaranteed, by phone and yes, we have, yes, which is quite often, at WhatsApp, so it’s actually like that, with my colleague who is sitting here now when she’s on late shift, she simply calls me again in the evening. So sometimes you’re actually already off work, but you’re always available (laughs). That is what you have in a small nursing service. You don’t go home and now it’s shift, so somehow you’re still there”*

(I3, A69–70)

##### Patients

When conducting and evaluating the interviews, it became clear that the patients play an important role in the category of social resources on the part of outpatient nursing staff. When asked about their motivation in their work, most outpatient nurses started by saying that joy in the eyes of the patients is the greatest appreciation and the greatest reward in their profession. The gratitude, joy, empathy, understanding, trust, and kind words of the patients are the reasons why they perform this job.

*“Yes, but immediately. You open the door at the customer, say a friendly good morning and a friendly smiling good morning comes up and says nicely that you are here. I don’t think there is any money in the world to make up for that. That is why I do it and why I want to do it. [...] the gratitude of these customers, these elderly people, simply this gratitude. And then I also know what I am doing this job for. Of course, the money is not unimportant, but that is the case. I like going to work in the morning and I look forward to it. [...] and not in the way I used to be in the restaurant business, where I would get up at some point in the morning and have a meatball in my stomach […]”*

(I1, A146–147)

#### 3.3.4. Coping Resources

The question of how workloads are dealt with or coped with includes aspects such as sporting activities, private activities, and exchanges with colleagues. However, the most important thing seems to be to allow time for oneself, to take care of oneself, and to say no. Being able to close the door behind you, in the truest sense of the word, is the most important thing. The handling of the associated stress factors simply must be learned in order to be able to carry out the job.

### 3.4. Wishes and Needs

At the end of the interview, the carers were asked about their wishes and needs regarding possible support, offers, and interventions. The needs range from a political to an individual level. After many outpatient care workers had emphasised their satisfaction, the need for support regarding physical requirements was primarily mentioned. The desire for higher appreciation by politicians in the form of higher wages and increased attractiveness of the profession, even more in response to the shortage of skilled workers, followed directly behind. This is one of the reasons why this occupation often does not seem attractive to young people.

*“I think that, politically speaking, there is a lot to come. In the health sector a lot would have to change, because [...], for the responsibility we have and what we take on during the day and on the patient, um, unfortunately we do not get paid for that and that is the only thing that bothers me about the whole thing. It is clear that my employer cannot do that, so what more should he pay if he doesn’t get anything from the health insurance company, that’s one thing that has to change at a higher level, it’s very urgent that the whole thing is recognised again. Because managers, they also have responsibility and they get paid for it, [...] if I make mistakes, then I have to answer for them and the responsibility, you just don’t get paid for it and that’s a pity, just a pity and that’s not the appreciation you would like to have (...) But well, it’s like this, we chose it (laughs) and can’t change it at the moment”*

(I5, A125–128)

Overall, however, it can be said that carers consider their work to be very meaningful. They love their work, and it is a vocation for them, which it should be. It is great fun for them. 

Figure 1 and Table 1 again provide an overview of the key findings. 

## 4. Discussion

This study examines current job demands, job resources, and the resultant strains experienced by outpatient caregivers in Germany. Relevant insights were gained into the working conditions of outpatient care workers in Germany. Outpatient care workers were faced with new and specific demands in their work. Social interactions with clients, communication with supervisors and colleagues, and the team spirit felt in the care services were important resources for their work. 

### 4.1. Job Demands

When looking at the results, it can be observed that the perceived work-related stress factors of outpatient nurses are very individual and differ from nurse to nurse. The explanation given in the background, that the subjective perception of stress is different for each person and therefore very individual, can be used for this [29,48].

This individuality is particularly evident in the psychological stress factors shown in the context of work organisation. This heterogeneity is expressed, apart from the individuals, in the consideration of the care services as such.

The time factor was emphasised in relation to work organisation, especially by the nursing staff. Time pressure is pointed out as a main stress factor in the studies demonstrated. It is striking, however, that the carers interviewed here did not allow themselves to be stressed by time. The nurses describe that this was felt only in the beginning when they started their profession. The GDA gives an indication of getting used to something if necessary and the long-term positive and negative consequences if the stresses persist. According to this definition, getting used to something can be seen as a kind of learning effect with a long-term, positive consequence [29,34]. 

The nurses’ statements about wanting to do even more for the patients can also be found in Büssing et al. (2000) [49] as such a burdening factor that they cannot realise their own demands based on the catalogue of services. However, in this study, the nursing staff emphasise the time factor rather than the catalogue of services. With the help of this aspect, however, it is possible to establish a link to work resources. Regarding resources and the feeling that the job is a vocation, loving the work and having fun with the patient can be strengthened. The ambulatory nurses give their patients total devotion, prioritising their needs before their own and thereby supporting the relevance of resources. However, this could lead to a vicious circle, as the interviewees explained that the most important thing is to take care of oneself.

On the level of work content, the interview partners primarily mention the challenging handling of personal fates and suffering and the resulting emotional demands on people. Nursing staff argue that this is particularly difficult to deal with, as they perform their work with a lot of passion. In addition, it is described that they never know what is going to happen, which is very demanding for them. This difficulty in planning the day results from the interaction work, as also mentioned by Böhle et al. [50]. However, the phenomenon of the learning effect must again be mentioned. The carers have become accustomed to it and have learned to deal with it or are trying to do so. The results show that relatives play an important role in the care of those in need, which supports the statements of Schneekloth [51] and others. Where people live and work together, differences of opinion and conflict situations arise. It is of great relevance on the part of the service provider to involve care-giving relatives more in the process and to strengthen their competence with the help of support services. According to Schneekloth [51], against the background of good care, relatives can be attributed a very important role or are already playing it.

In the context of the working environment, the physical factors and the resulting heavy physical work on the part of carers are most frequently mentioned, reinforcing the statements of, among others, Büssing et al. [49], Schilgen et al. [52], and Weyerer et al. [53]. The interviewees are concerned that they do not know whether they will still be able to perform the work physically in old age due to strength reasons. Without this challenge, they could imagine carrying out the job all their lives. The fact that respondents from one care service mention different perceptions of physical strain indicates that further research should again primarily take the operational perspective. 

Moreover, Bleses and Busse [54] show that a stronger focus on new technologies in the context of digitisation adapted to the needs of carers (e.g., industry software on smartphones) is of high relevance. As mentioned by Bleses and Busse [54], the nurses interviewed in this study are less tangentially affected by new technologies and the associated challenges. This topic was only touched on by one nurse, who reported that it is perceived positively since in the software on mobile phones, time for breaks can be entered and thus taken. However, the rapid developments in the context of digitisation show that future research and adequate consideration of the new technologies need to be conducted in this professional field. 

### 4.2. Job Resources

The study findings show that the work resources of outpatient care workers become visible on different levels—on individual, organisational, and social levels. All in all, when interpreting the work resources, it is noticeable that in their descriptions, the nursing staff very often drew comparisons with other jobs (e.g., in other outpatient and inpatient care services) in which they have already worked. They described that they have had worse experiences with previous employers. It was observed that, in the same breath, they underpinned their current resources and strengthened their current work as an outpatient nurse and job with this comparison. A study by Wirth et al. [55] could also provide an explanation, at least for the comparisons with inpatient care, as their results show that employees in inpatient care have more stress factors. Büssing et al. [49] also confirm the moderate levels of stress in outpatient care. At the same time, however, they warn against the high levels of stress and strain in between. Thus, their appeal not to lose sight of the need for outpatient care in any way can be endorsed in this paper.

During the analysis of the results, it became clear that carers cite a variety of work resources at all levels. Autonomy, flexibility, quiet working, own work rhythm, separation, support from the company, exchange with colleagues, and appreciation from patients are some examples. It is striking that the aspect of gratitude of the patients was emphasised as the biggest driving factor for all, which is particularly emphasised in this study compared to previous studies.

However, regarding personal resources, the love of the profession of outpatient nursing and the knowledge of their vocation are usually mentioned first. The explanation that the profession must be loved to be able to carry it out could explain why it seems that during the analysis, the stress factors tended to recede into the background. Independent, autonomous, flexible, and free work is also an important resource, as Bleses and Jahns [6,14] found out in their studies. Nursing staff enjoy the care at home and the cooperation with each other—especially in comparison to the work in inpatient facilities. The interview partners reported that they are fully available for the customers at that moment, without any disturbance. Bleses and Jahns [6,14] support these results in their investigations.

The conclusions drawn by Maurits et al. [20] that a higher feeling of self-perceived autonomy leads to a higher work commitment and less fluctuation among the nursing staff confirm the results. The spatial and temporal flexibility were described by the interviewees as a resource rather than a burden. It should therefore be emphasised that some factors function as a work resource rather than a workload for the interviewees, to which the explanations of Lazarus and Folkman [48] regarding the different perceptions of stress can be referred once again.

In the context of work-related organisational resources, particular attention was paid to the appreciation of the company. The interviewees described that great care is taken to ensure that they are well, and the contact is thus perceived as very human and pleasant. Company celebrations, further training, handover regulations, open communication structures and a culture of criticism, tour planning, service meetings, a good familiarisation period, no time reductions in the form of, e.g., overtime capping, support in all respects and especially in emergencies, possibilities of expressing wishes, and sufficient compensation possibilities all act as resources.

On a social level, colleagues and superiors often function as a work resource for the carers. In this respect, differences can be drawn compared to the studies by Glaser and Höge [56]. In these studies, the danger of social isolation is mentioned since ambulatory care workers mainly work alone, and thus social exchange is less possible. This statement cannot be confirmed in any way by the data analysis of this present study. On the other hand, when looking at the results, it becomes clear that the gratitude of the patients, the joy in the eyes of the patients, and their kind words are another, possibly even the most important, working resource on a social level. This is particularly noticeable when looking at the results. This meaningfulness can also be observed in the results of the analysis of Bleses and Jahns [6,14], which result from the interaction work. The thesis is that people’s joy and gratitude are so predominant that they feel the burden is less heavy, or that they feel that they have to cope with the stress or have already become so accustomed to it that they do not notice it.

Social support and appreciation, as already emphasised in the literature [57,58], can be found in the working situations of the interviewees. The results could be used to support this aspect of a significant predictor of the work commitment of carers.

The results presented in this paper indicate that the work motivation of the interviewed nurses is particularly initiated by the gratitude of the patients. This derivation can explain the fact that the work demands can be compensated by the work resources, e.g., by the apparently most serious resource: the gratitude of the patients.

### 4.3. Strengths and Limitations

At this point, it should be pointed out once again that there is no claim to generalizability. The reason for this is the explorative research design of this study. This study contributes to a better understanding of the current work-related mental stress factors and work resources of outpatient nursing staff. It can serve as a good basis for further research. By conducting a personal interview, the questionnaire used cannot be filled out at home, which may be a barrier for reasons of convenience, but misunderstandings and questions can be clarified directly. In addition, a personal interview requires more resources in comparison, but gestures and facial expressions are omitted, which means that important additional information can be gathered [59].

The choice of the care services could represent a limitation. It could be assumed that only those nursing services that already have a wide range of health promotion measures, e.g., within the framework of occupational health management, have given their consent to an interview. Since contact with the nursing services was usually established via nursing service management, with the help of the so-called “gatekeeper principle”, the latter usually made a pre-selection of the interview partners and allocated them directly. This meant that the participants themselves were often not able to decide whether to participate. It also happened that the nursing services communicated a direct rejection of the research project without leaving the decision to the staff.

In the framework of the results, some overlap of individual statements between the categories can be observed. These could not always be clearly separated from each other. This is also the reason for the joint consideration of work resources and coping strategies. It should also be noted that although some carers work in the same care services, there is the possibility of different statements. This research project is primarily concerned with subjective perceptions rather than detailed comparisons and analyses of companies. Nevertheless, these comparisons are not excluded but are largely neglected in the context of the work. Regarding this subjective perspective, many commonalities as well as individual perceptions, views, and needs can be identified among the nursing staff in all categories.

However, enough participants were recruited, which is why the recruitment of outpatient nursing staff can be assessed as successful, even if no second wave of interviews took place. Regarding the selection of the target group, the criterion of sufficient German language skills represents a limitation. Non-German-speaking nurses could have added further relevant information. However, the international context could not be considered here due to limited personnel resources and the different objectives of this work. Nor could a balanced gender ratio be achieved regarding the study population. The gender distribution in this occupational field could be the reason for this. However, it should be emphasised as a strength that interview partners of all age groups with different school qualifications were recruited and recorded. Additionally, during the interviews, continuous quality control can be observed and can be named as a strength, because the guideline was continuously adapted. The quality of this qualitative study was assessed according to the seven quality criteria of Steinke (2005): intersubjective traceability, indication of the research process, empirical anchoring, limitations, coherence, relevance, and reflected subjectivity [60]. The discussion of these revealed that the best possible result was achieved in view of the available resources.

### 4.4. Future Research and Practical Implications

The discussion of the results of this study shows that future in-depth research in this field is essential. It should be extended to other regions to be able to draw a comprehensive picture of the current mental workload and resources of outpatient nursing staff, their working conditions, and especially the consequences of stress and thus the health of the staff, and to generalise the results. The quality criterion of generalizability could then be fulfilled. The results from the literature to date and the further component created here—the present study—can be used as a starting point for this. By means of larger and randomised samples as well as quantitative analyses with answer scales in standardised surveys, the connections explained in this work and especially in the discussion could be confirmed, further factors identified, differences shown in existing literature illuminated, and aspects reformulated into measurable variables. The emphasised differences in the perception of stress and the associated individuality and heterogeneous reactions of employees should be taken into consideration. The presented study could gain a wide range of additions and a broader perspective through all future research. The cycles shown in the JD-R model should also be examined in the same breath using quantitative survey methods in the working context of outpatient care to be able to make specific statements about the interactions. It is particularly recommended to focus on the extent of learning and habituation effects to see to what extent those aspects still act as a burdening and challenging factor and determine the daily routine of the nursing staff or not.

In the entire research project, it should be noted that outpatient nursing staff are of great importance, but that a comprehensive approach must also consider the management positions of a nursing service, such as management and nursing service management. It would also be a further consideration to survey only career starters in one block in order to obtain a reflection of the often-mentioned challenging beginning of the job. Even if women dominate the profession of outpatient nursing, it would be desirable for future research to gain more men as interview partners to be able to generate an even more comprehensive picture.

The fact that this study has focused primarily on the subjective perceptions and individual needs of outpatient nursing staff means that it can be assumed that it is in everyone’s interest to broaden the view in the future beyond the individual to the company and interpersonal level in order to identify specific burdens. The reason for this is the indications given by the interviewees about the apparent differences between companies regarding working conditions, which they have already experienced firsthand. At the same time, inpatient care should not be pushed into the background, and the comparison with outpatient care should be pursued further [49,55].

In addition to the implications for research, the following practical implications can be derived, and recommendations for action can be generated. The presented results could be a basis and support for the target-group-specific development of necessary health promotion measures in the workplace setting to keep outpatient nursing staff healthy and in their jobs in the long term. The special setting of this work requires special attention in the context of health promotion measures at the behavioural and relational levels [15]. This therefore means the synthesis of employees and employers in designing a healthy workplace [30,49] in the form of creating a balance between work demands and resources in the workplace [61]. The question is also how these can be effectively interlinked [30]. Demerouti and Nachreiner (2019) also describe the conclusions for practice using the JD-R model. Employers should offer their employees sufficient work resources and affordable work requirements [25].

A study by Schmidt and Diestel (2013), which points out the flexibility of coping, i.e., active coping, should also be mentioned here. The use of a variety of coping strategies and not a rigid application of a style is the means of choice to increase the effectiveness of the use of personal resources [62]. Employees should be strengthened in their convictions about what they can achieve with the skills they already have.

Approaches at the institutional level can be based on the organisational aspects outlined, such as the elimination of organisational deficiencies in the form of fixed-duty roster regulations. At the same time, organisational resources should be further strengthened (e.g., provision of aid). A general appreciation in the form of support in all matters mentioned in this paper (e.g., care, communication structures, and respectful treatment) should be considered as a higher priority. The initial approaches to design needs already mentioned by Bleses and Jahns [6], Rickard et al. [17], and Wirth [55] could be considered or linked to them. A higher level of reliability regarding duty roster compliance could increase job satisfaction and motivation [6]. 

Furthermore, to facilitate a better processing of the personal fates of patients and to strengthen the team internally, employee cafés could be introduced. This would enable and guarantee even more intensive communication between the employees, as the carers describe the exchange with their colleagues and their support as very valuable. The idea of external moderation could be considered in this context.

The revision of the training model could also be considered as a further approach. Based on the analyses in the discussion, training could become an elementary component in the development of suitable measures. Case studies could be used to prepare nurses even better for their work in different situations and to strengthen the associated competencies.

In the next step, larger companies should not be disregarded, as it can be assumed that there is a need for support measures on the part of the outpatient nursing staff in these companies as well.

Furthermore, when developing workplace health promotion measures, attention should be directed to the behavioural level. Here, measures can start with personal and social resources as well as coping strategies to increase the commitment to work. The love of the profession, the gratitude of the patients, and the need for nurses to care for themselves are to be mentioned in this context. The continuous promotion of health literacy of nurses on an individual level and the further development of concepts regarding this can also be mentioned. One idea of implication here would be mindfulness training and the strengthening of self-management skills. A reduction in stress at the individual level can be achieved by the individual learning to cope with the stress [15,62].

Subsequently, however, the social level must also be involved. The aspects mentioned above and the potential for improvement of the interview partners in the category “wishes and needs” can serve as starting points and can be identified as central design requirements. To attract and retain sufficient, healthy, and motivated young employees in the long term, it could be assumed that a comprehensive change is needed, as previous statements from previous years have shown. A fundamental increase in the attractiveness of the profession in the form of higher wages and more staff (and thus the elimination of stagnating staffing levels), the health-promoting working conditions already mentioned, and a better work–life balance were still identified by the interviewees as fixed elements and must not be ignored, as reported in the Nursing Report from 2019 [18]. Exclusively selective measures are not sufficient [5], which can be confirmed by the present study.

## 5. Conclusions

In summary, it can be said that many psychological stress factors could be identified in outpatient nursing staff in Germany, which can, however, be compensated by various identified work resources at all levels.

Time pressure, dealing with the patients’ personal fates, and physical demands were identified as stress factors. The patients’ gratitude, the company’s appreciation, and the love of the profession are the main resources. Even if the stress factors appear to be largely similar to the existing literature and a large number of them are already known, they could be observed to varying degrees, and especially in different interrelationships with work resources and coping strategies. Taking care of oneself and creating a sufficient balance in the private environment are examples of coping strategies. 

This study offers valuable insights into the working world of outpatient care, with a special focus on work resources and coping strategies. The JD-R model proves to be a helpful theoretical framework for establishing the relationships between work demands and resources as well as coping strategies. Even if carers assess their mental health as good in a professional context, it is important to note here that this is a snapshot and not a claim to generalisation. These results serve to highlight gaps in research that still exist and to focus further particularly quantitative research to be able to measure the extent of the individual factors, to have the results confirmed, and to strengthen connections and pursue the goal of generalisability. The present study could gain a variety of additions and a further perspective through all future research. In addition, it is important to take adequate account of the development of digitisation and the associated potential work requirements in the field of outpatient care. 

It is of high relevance to secure and promote the health of outpatient nursing staff to maintain and strengthen their work motivation and thus cover the need for care at the same time. This can hopefully stimulate research and practice to constantly shape the further development of health-promoting measures in the context of workplace design so that outpatient nursing staff can pursue their vocations for as long as possible.

## Figures and Tables

**Table 1:** Overview of the main results of the interviews (source: own illustration).

**Personal Resources**
Love for the profession and fun at work The profession is a vocation Finding the work enjoyable Independent and autonomous work, flexibility, and freedom Sensory experience Great interest in gaining new knowledge and keeping up to date with the latest information Clear expression of wishes and needs Keeping calm Taking on additional tasks in the office (e.g., music and dancing) Being self-observant and creating compensations Saying no and drawing boundaries e.g., they can distance themselves from personal destinies or try to do so ▪ Car as a resting place and breaks
**Psychosocial Outcomes**
The nurses are very satisfied with their job. They experience a sense of meaning in their work. They love their work very much. The profession is a vocation for them. The gratitude of the people is the most valuable thing about it. Over time, they have become accustomed to many things (especially the stress factors) and have learned to deal with them.
**Wishes and needs**
Salary increase Counteracting the shortage of skilled workers Less unexpected call-ins Reducing overtime Increasing the attractiveness of the profession Support in the form of, e.g., aid, as the physical stress factor is weighted most heavily

## Data Availability

The data analysed during the current study are not publicly available due to German national data protection regulations. They are available on individual request from the corresponding author.

## References

[1] Nowossadeck S. (2013). Demografischer Wandel, Pflegebedürftige und der künftige Bedarf an Pflegekräften. Eine Übersicht. Bundesgesundheitsblatt Gesundheitsforsch.Gesundheitsschutz.

[2] Pick P., Brüggemann J., Grote C., Grünhagen E., Lampert T. (2004). Pflege: Schwerpunktbericht zur Gesundheitsberichterstattung des Bundes.

[3] Rothgang H., Müller R., Unger R. (2013). Schwerpunktthema: Reha bei Pflege. BARMER GEK Pflegereport 2013: Schriftenreihe zur Gesundheitsanalyse.

[4] Kuhlmey A., Blüher S., Schaeffer D., Wingenfeld K. (2014). Demografische Entwicklung in Deutschland—Konsequenzen für Pflegebedürftigkeit und pflegerische Versorgung. Handbuch Pflegewissenschaft: Studienausg.

[5] Jacobs K., Kuhlmey A., Greß S., Klauber J., Schwinger A. (2019). Pflege-Report 2019: Mehr Personal in der Langzeitpflege—Aber Woher?.

[6] Bleses P., Jahns K., Becke G., Bleses P. (2015). Neugestaltung der Koordination und Interaktion in der ambulanten Pflege: Chancen und Anforderungen für Führungs- und Pflegekräfte. Interaktion und Koordination: Das Feld Sozialer Dienstleistungen.

[7] Büscher A., Schaeffer D., Wingenfeld K. (2014). Ambulante Pflege. Handbuch Pflegewissenschaft: Studienausg.

[8] Schaeffer D., Wingenfeld K. (2014). Handbuch Pflegewissenschaft. Studienausg.

[9] Techniker Krankenkasse Menschen Wollen zu Hause Gepflegt Werden. https://www.tk.de/presse/themen/pflege/pflegende-angehoerige/pflegewuensche-meinungspuls-pflege-2042956.

[10] Bundesministerium für Gesundheit Online-Ratgeber Pflege: Pflegedienst und Pflegesachleistungen. https://www.bundesgesundheitsministerium.de/pflegedienst-und-pflegesachleistungen.html.

[11] Bundesanstalt für Arbeitsschutz und Arbeitsmedizin Arbeit in der Pflege—Arbeit am Limit?. Arbeitsbedingungen in der Pflegebranche: BIBB/BAuA-Faktenblatt 10..

[12] Fuchs M., Weyh A. (2013). Auswirkungen des demografischen Wandels auf die Fachkräftesituation im Pflegebereich in Mitteldeutschland. Eine Analyse für Sachsen, Sachsen-Anhalt und Thüringen. Bundesgesundheitsblatt Gesundheitsforsch. Gesundheitsschutz.

[13] Barthelme G., Garms-Homolová V., Schmidt R., Entzian H., Giercke K.-I., Klie T. (1999). Belastungen in der ambulanten Pflege. Die Versorgung Pflegebedürftiger alter Menschen in der Kommune: Daseinsvorsorge, Leistungserbringung und bürgerschaftliche Verantwortung in der Modernisierung der Pflege.

[14] Bleses P., Jahns K., Becke G., Bleses P., Frerichs F., Goldmann M., Hinding B., Schweer M.K.W. (2016). Soziale Innovationen in der ambulanten Pflege. Zusammen—Arbeit—Gestalten: Soziale Innovationen in sozialen und Gesundheitsbezogenen Dienstleistungen.

[15] Zimber A., Haberstroh J., Pantel J. (2011). Belastungen, Ressourcen und Beanspruchung in der Altenpflege. Demenz Psychosozial Behandeln: Psychosoziale Interventionen bei Demenz in Praxis und Forschung.

[16] Techniker Krankenkasse Gesundheitsreport: Pflegefall Pflegebranche? So Geht’s Deutschlands Pflegekräften. https://www.tk.de/resource/blob/2059766/2ee52f34b8d545eb81ef1f3d87278e0e/gesundheitsreport-2019-data.pdf.

[17] Rickard G., Lenthall S., Dollard M., Opie T., Knight S., Dunn S., Wakerman J., MacLeod M., Seller J., Brewster-Webb D. (2012). Organisational intervention to reduce occupational stress and turnover in hospital nurses in the Northern Territory, Australia. Collegian.

[18] Bundesministerium für Gesundheit Gesundheitsförderung für Pflegekräfte: Wer Pflegt die Pflege?: Ausgangslage: Die Arbeitssituation in der Pflege. https://www.bundesgesundheitsministerium.de/fileadmin/Dateien/5_Publikationen/Pflege/Praxisseiten_Pflege/10.0.1_Service_Material.pdf.

[19] Grabbe Y., Nolting H.-D., Loos S., Krämer K. (2006). DAK-BGW-Gesundheitsreport 2006 Ambulante Pflege: Arbeitsbedingungen und Gesundheit in Ambulanten Pflegediensten.

[20] Maurits E.E.M., de Veer A.J.E., van der Hoek L.S., Francke A.L. (2015). Autonomous home-care nursing staff are more engaged in their work and less likely to consider leaving the healthcare sector: A questionnaire survey. Int. J. Nurs. Stud..

[21] Diehl E., Rieger S., Letzel S., Nienhaus A., Escobar Pinzon L.C. (2019). Belastungen und Ressourcen von Pflegekräften der spezialisierten Palliativversorgung. Pflege.

[22] Vu-Eickmann P., Loerbroks A. (2017). Psychosoziale Arbeitsbedingungen Medizinischer Fachangestellter: Ergebnisse einer qualitativen Studie zu den berufsspezifischen Belastungen, Ressourcen, Präventionsmöglichkeiten und Interventionsbedürfnissen. Z Evid Fortbild Qual Gesundhwes.

[23] Bakker A.B., Demerouti E. (2007). The Job Demands-Resources model: State of the art. J. Manag. Psychol..

[24] Bakker A.B., Demerouti E., Cooper C.L. (2014). Job Demands-Resources Theory. Wellbeing.

[25] Demerouti E., Nachreiner F. (2019). Zum Arbeitsanforderungen-Arbeitsressourcen-Modell von Burnout und Arbeitsengagement—Stand der Forschung. Z. Arbeitswissenschaft.

[26] Demerouti E., Bakker A.B., Nachreiner F., Schaufeli W.B. (2001). The job demands-resources model of burnout. J. Appl. Psychol..

[27] Hakanen J.J., Schaufeli W.B., Ahola K. (2008). The Job Demands-Resources model: A three-year cross-lagged study of burnout, depression, commitment, and work engagement. Work Stress.

[28] Bakker A.B., Demerouti E. (2017). Job demands-resources theory: Taking stock and looking forward. J. Occup. Health Psychol..

[29] Gemeinsame Deutsche Arbeitsschutzstrategie Arbeitsschutz in der Praxis. Psychische Arbeitsbelastung und Gesundheit..

[30] Morschhäuser M., Ertel M., Lenhardt U. (2010). Psychische Arbeitsbelastungen in Deutschland: Schwerpunkte—Trends—Betriebliche Umgangsweisen. WSI.

[31] (1996). Ergonomische Grundlagen Bezüglich Psychischer Arbeitsbelastung, Teil 1: Allgemeines und Begriffe.

[32] Gemeinsame Deutsche Arbeitsschutzstrategie Leitlinie Beratung und Überwachung bei psychischer Belastung am Arbeitsplatz. http://www.gda-portal.de/DE/Downloads/pdf/Leitlinie-Psych-Belastung.pdf?__blob=publicationFile&v=5.

[33] Schüle E., Psychische Belastungen vs Beanspruchung. http://dg-pg.de/_wordpress/wp-content/uploads/2014/08/20120411-Sch%C3%BCle_-Belastung-vs-Beanspruchung.pdf.

[34] Kunz T. Belastungs- und Beanspruchungsmodell: Gemeinsame Deutsche Arbeitsschutzstrategie. https://www.gda-psyche.de/DE/Zahlen-Daten-Fakten/Entstehungsmodelle/Belastungs-und-Beanspruchungsmodell/inhalt.html.

[35] Schubert F.-C., Knecht A. (2015). Ressourcen—Merkmale, Theorien und Konzeptionen im Überblick: Eine Übersicht über Ressourcenansätze in Soziologie, Psychologie und Sozialpolitik.

[36] Willutzi U., Röhrle B. (2008). Klinische Ressourcendiagnostik. Lehrbuch der Klinisch-Psychologischen Diagnostik.

[37] Nowossadeck E., Demografische Alterung und Folgen für das Gesundheitswesen GBE Kompakt 2012. https://www.rki.de/DE/Content/Gesundheitsmonitoring/Gesundheitsberichterstattung/GBEDownloadsK/2012_2_Demografischer_Wandel_Alterung.pdf?__blob=publicationFile.

[38] Döring N., Bortz J. (2016). Forschungsmethoden und Evaluation in den Sozial- und Humanwissenschaften.

[39] Helfferich C. (2004). Die Qualität Qualitativer Daten.

[40] Helfferich C. (2011). Die Qualität Qualitativer Daten.

[41] Kruse J., Weber K.-M., Dresing T., Pehl T., Schmieder C. (2015). Qualitative Interviewforschung: Ein Integrativer Ansatz. 2., Überarbeitete und Ergänzte Auflage.

[42] Fischmann W., Netzwerke zur Gesundheitsförderung für KMU ASU Zeitschrift für Medizinische Prävention 2019. https://www.asu-arbeitsmedizin.com/schwerpunkt/netzwerke-zur-gesundheitsfoerderung-fuer-kmu.

[43] Sommer D., Kuhn D., Sommer D. (2004). Betriebliches Gesundheitsmanagement in kleinen und mittleren Unternehmen. Betriebliche Gesundheitsförderung: Ausgangspunkte—Widerstände—Wirkungen.

[44] Flick U. (2019). Qualitative Sozialforschung: Eine Einführung: Völlig Überarb.

[45] Kuckartz U. (2016). Qualitative Inhaltsanalyse: Methoden, Praxis, Computerunterstützung.

[46] Pawicki M., Kuckartz U. (2014). Qualitative Inhaltsanalyse. Methoden, Praxis, Computerunterstützung. J. Educ. Res. Online.

[47] Dresing T., Pehl T. (2017). Praxisbuch Interview, Transkription & Analyse: Anleitungen und Regelsysteme für Qualitativ Forschende.

[48] Lazarus R.S., Folkman S. (1984). Stress, Appraisal, and Coping.

[49] Büssing A., Giesenbauer B., Glaser J., Höge T. (2000). Ambulante Pflege: Arbeitsorganisation, Anforderungen und Belastungen: Eine Pilotstudie mit Erfahrungsberichten.

[50] Böhle F., Stöger U., Weihrich M. (2015). Interaktionsarbeit Gestalten: Vorschläge und Perspektiven für Humane Dienstleistungsarbeit.

[51] Schneekloth U. (2006). Entwicklungstrends und Perspektiven in der häuslichen Pflege. Zentrale Ergebnisse der Studie Möglichkeiten und Grenzen selbständiger Lebensführung (MuG III). Z. Gerontol. Geriatr..

[52] Schilgen B., Handtke O., Nienhaus A., Mösko M. (2019). Work-related barriers and resources of migrant and autochthonous homecare nurses in Germany: A qualitative comparative study. Appl. Nurs. Res..

[53] Weyerer S., Schäufele M., Anton R., Teufel S., Sattel H. (2001). Gesundheitsrisiken in Ambulanten Pflegediensten: Abschlussbericht an die Berufsgenossenschaft für Gesundheitsdienst und Wohlfahrtspflege.

[54] Bleses P., Busse B., Bleses P., Busse B., Friemer A. (2020). Digitalisierung der Pflegearbeit in der ambulanten Pflege: Herausforderungen und Gestaltungsmöglichkeiten guter Arbeitsqualität. Digitalisierung der Arbeit in der Langzeitpflege als Veränderungsprojekt.

[55] Wirth T., Ulusoy N., Lincke H.-J., Nienhaus A., Schablon A. (2017). Psychosoziale Belastungen und Beanspruchungen von Beschäftigten in der stationären und ambulanten Altenpflege. Arbeitsmed. Sozialmed. Umweltmed..

[56] Glaser J., Höge T. Probleme und Lösungen in der Pflege aus Sicht der Arbeits- und Gesundheitswissenschaften. https://www.baua.de/DE/Angebote/Publikationen/Berichte/Gd18.html.

[57] García-Sierra R., Fernández-Castro J., Martínez-Zaragoza F. (2016). Relationship between job demand and burnout in nurses: Does it depend on work engagement?. J. Nurs. Manag..

[58] Trybou J., Germonpre S., Janssens H., Casini A., Braeckman L., de Bacquer D., Clays E. (2014). Job-related stress and sickness absence among belgian nurses: A prospective study. J. Nurs. Scholarsh..

[59] Schulz M., Ruddat M. (2012). “Let’s talk about sex!” Über die Eignung von Telefoninterviews in der qualitativen Sozialforschung. Forum Qualitative Sozialforschung. Forum Qual. Soc. Res..

[60] Steinke I., Flick U., Kardorff E., Steinke I. (2012). Gütekriterien qualitativer Forschung. Qualitative Forschung: Ein Handbuch.

[61] LeGal P., Rhéaume A., Mullen J. (2019). The long-term effects of psychological demands on chronic fatigue. J. Nurs. Manag..

[62] Schmidt K.-H., Diestel S. (2013). Job demands and personal resources in their relations to indicators of job strain among nurses for older people. J. Adv. Nurs..

